# Austerity policy and child health in European countries: a systematic literature review

**DOI:** 10.1186/s12889-020-08732-3

**Published:** 2020-05-19

**Authors:** Luis Rajmil, Anders Hjern, Nick Spencer, David Taylor-Robinson, Geir Gunnlaugsson, Hein Raat

**Affiliations:** 1 Retired, paediatrician and public health and epidemiology specialist, Homer 22 1rst 1, Barcelona, Spain; 2 Centre for Health Equity Studies (CHESS), Stockholm University/Karolinska Institutet, SE-106 91 Stockholm, Sweden; 3 Emeritus Professor of Child Health, Division of Mental Health and Wellbeing, Warwick Medical School University of Warwick, Coventry, CV4 9JD UK; 4 Clinical Senior Lecturer, Department of Public Health and Policy, Whelan Building University of Liverpool, Liverpool, UK; 5 Faculty of Sociology, Anthropology and Folkloristics, University of Iceland, Reykjavik, Iceland; 6 Department of Public Health, Erasmus Medical Center, University Medical Center, PO Box 2040, 3000 CA Rotterdam, The Netherlands

**Keywords:** Austerity, Child health, Child poverty, Economic crisis, Great recession, Social inequalities

## Abstract

**Background:**

To analyse the impact of austerity measures taken by European governments as a response to the 2008 economic and financial crisis on social determinants on child health (SDCH), and child health outcomes (CHO).

**Methods:**

A systematic literature review was carried out in Medline (Ovid), Embase, Web of Science, PsycInfo, and Sociological abstracts in the last 5 years from European countries. Studies aimed at analysing the Great Recession, governments’ responses to the crisis, and its impact on SDCH were included. A narrative synthesis of the results was carried out. The risk of bias was assessed using the STROBE and EPICURE tools.

**Results:**

Fourteen studies were included, most of them with a low to intermediate risk of bias (average score 72.1%). Government responses to the crisis varied, although there was general agreement that Greece, Spain, Ireland and the United Kingdom applied higher levels of austerity. High austerity periods, compared to pre-austerity periods were associated with increased material deprivation, child poverty rates, and low birth weight. Increasing child poverty subsequent to austerity measures was associated with deterioration of child health. High austerity was also related to poorer access and quality of services provided to disabled children. An annual reduction of 1% on public health expenditure was associated to 0.5% reduction on Measles-Mumps-Rubella vaccination coverage in Italy.

**Conclusions:**

Countries that applied high level of austerity showed worse trends on SDCH and CHO, demonstrating the importance that economic policy may have for equity in child health and development. European governments must act urgently and reverse these austerity policy measures that are detrimental to family benefits and child protection.

## Background

The Great Recession was characterised by an economic and financial crisis following the bail out of the banks in 2008. There followed a decline in gross domestic product (GDP) and high levels of unemployment, affecting European countries and worldwide [1]. Nevertheless, the magnitude of the crisis, its size, duration and geographical spread was different across European countries. Some countries experienced more substantial and sustained falls in GDP and rises in unemployment than others.

From 2010 to 11 onwards most European countries adopted austerity measures in response to the recession. The term austerity refers to measures taken by Governments to approach deficit reduction strategies following the financial crisis of 2008, primarily though limits to government expenditures and tax increases, and independently on the methods used to assess it. These austerity measures taken by governments, mainly characterised by reducing social spending and increasing taxation, were neither homogeneous nor similarly implemented; some protected public sector programs and systems while others instituted large budget cuts in education, health, and other public services [2].

The impact of the economic crisis on child health has been widely analysed. Studies suggest that the economic crisis disproportionately affected the most vulnerable groups, mainly children and youth from disadvantaged families [3]. Most studies of the impact of economic crisis on health have not distinguished between economic crises themselves and policy responses to these crises [4]. Although it would be difficult to disentangle the impact of the crisis itself from the Governments’ responses [5], some studies have attempted to separately analyse this effect on the general population, mainly addressed specifically at adult health [6]. Less attention has been addressed to the impact of austerity measures on social determinants of child health (SDCH) and child health outcomes (CHO) [7, 8].

Childhood is a vulnerable period to the main determinants of health. Factors related to financial, human and social capital have potential influence on future child health and development [9, 10]. Moreover, there is evidence for the profound effects of social factors on health throughout childhood and into adulthood [11–13].

The objective of this systematic literature review was to analyse the impact of austerity measures taken by European governments as a response to the 2008 economic and financial crisis on the SDCH and CHO. The hypothesis was that a higher level of austerity could be associated with a greater impact on inequalities and health, especially in the childhood population, where investment in the early lifecourse is essential to facilitate equitable and adequate child development, as well as the future adult health.

## Methods

A systematic literature review was carried out in the databases Medline (Ovid), Embase, Web of Science, PsycInfo, and Sociological abstracts. Manual search for local published and unpublished documents were also included.

The Preferred Reporting Items of Systematic reviews Meta-Analyses (PRISMA, http://www.prisma-statement.org/) guidelines was followed, although some items are not applicable given the characteristics of included studies. Search strategy adapted to each Database analysed included the terms “austerity”, “financial crisis”,” downturn”, “child health outcomes”, “child health inequalities”, and “healthcare services” (search strategy available in the appendix).

### Inclusion criteria

Included studies were those published in the last 5 years (from January 1rst, 2014 to November 4th, 2019) that reported on the impact of changes in family/children benefits, or in budgets, or access to healthcare system, or to social benefits as Government responses to the Great Recession on child health and living conditions (children <18y) in European countries. Studies should include the results of at least one European country, at least one SDCH or CHO, separately from adult outcomes. Studies should analyse austerity and/or government responses to the crisis as the main exposure measure. Systematic or narrative reviews, longitudinal, before-after, qualitative and cross-sectional studies were included.

### Exclusion criteria

Those studies reporting only the impact of the crisis were excluded, except when it could be linked to changes in policy responses; general population studies without stratification by age group or studies of adult populations, and Editorial or opinion papers were also excluded.

### Procedures

Abstracts obtained by the initial search strategy were assessed for possible inclusion by two of the authors (AH, LR). Full text papers of the studies were obtained in doubtful cases and were independently assessed by these authors. Differences of opinion on inclusion were decided by discussion and consensus among all authors.

### Data extraction and analysis

Data extracted included: *setting* (according to the country: international, national or regional study); *type of study* (trends of repeated cross-sectional data, before-after comparative study, cross-sectional study, review, etc); *objective of study*; *years covered by the study*; the specific *target population* (age range); *measures of exposure,* including austerity measures; *outcome measures;* and results in terms of *impact on SDCH and/or CHO*.

The risk of bias of included studies was assessed by 4 of the authors (AH, DTR, NS, LR) using the Strengthening the Reporting of Observational Studies in Epidemiology (STROBE) initiative for quantitative studies [14]. Qualitative studies were assessed using EPICURE (Engagement, Processing, Interpretation, Critique Usefulness, Relevance and Ethics) [15]. STROBE items not applicable in certain study designs due to the inclusion of indicators based on ecological data (i.e., items related to characteristics of the individuals included in these indicators) were excluded from the evaluation of these specific studies. Thus, the average score between evaluators was calculated and subsequently the percentage achieved by each study on the total items evaluated. Risk of bias was stratified as high (< 50%), intermediate (50–75%) and low (>75%). For qualitative studies, 4 out of the 7 items (P, I, U, R) were considered essential to assess studies as average or low risk of bias (the latter if also met at least one of the other items). The lack of one or more of these 4 essential items was considered as high risk of bias. Analysis was stratified regarding the type of studies, the exposure measure of austerity, and the main outcome measures (SDCH, [CHO], and healthcare, and preventive services). A meta-analysis was not carried out given the nature of the study design and heterogeneity of the findings. A narrative synthesis of the results was carried out.

## Results

**Fig. 1:**
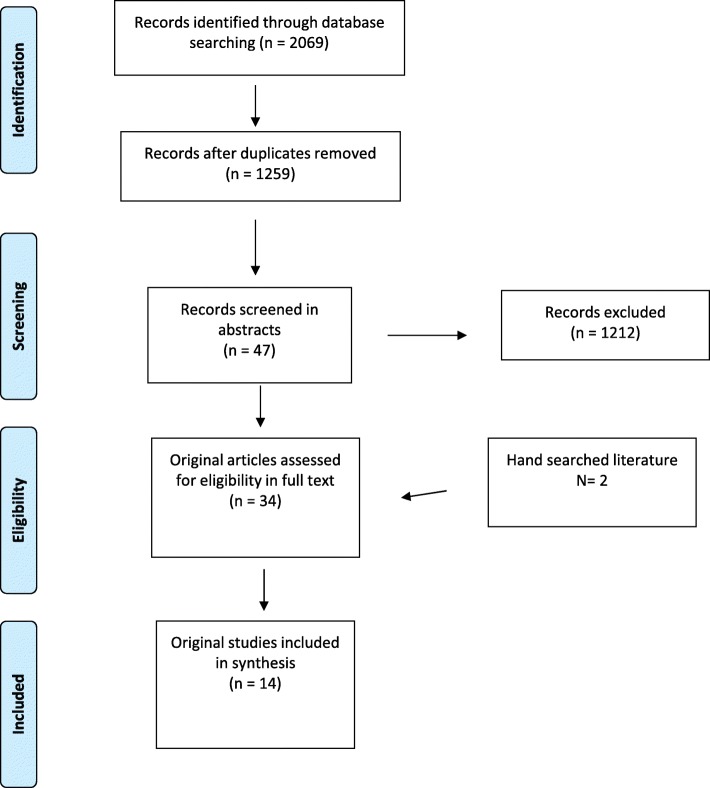
Search flow

Figure 1 shows the results of the literature search; 14 studies met the inclusion criteria.

### Characteristics of studies and exposure measures

Table 1 spells out the characteristics of included studies: *study design* (cross-sectional, trends over time, before-after approach, cohort study, or qualitative study), *country* (individual country, or the number of countries analysed), *exposure* (type of austerity measure and source of data), *outcome* (SDCH and/or CHO), and the *risk of bias*. Nine studies report time trend analyses of cross-sectional repeated data and two studies report before-after analyses following the implementation of austerity measures. The level of austerity measures was assessed by methods proposed by the International Monetary Fund (IMF) [21], the European Union’s Maastricht criteria [16], or by analysing spending on families/children social protection in specific periods of time [17, 20].

**Table 1:** Type of studies and risk of bias

Type of study (first author, year)	Country/−ies	Exposure measure (year/s of study, source of data)	Outcome measure	Risk of bias (% of the total score-STROBE)
				Average score = 72.1
**Cross-sectional**				
Horridge et al. 2019 [16]	32 EU countries	Survey to professionals and families/children. Countries classified according to the level of austerity following the European Union’s Maastricht criteria (2016–17)	Healthcare services to disabled children. Requests on changes in the quality and characteristics of services in the last years	Intermediate (70.4)
**Trends over time - Repeated cross-sectional analysis**				
Chzhen et al. 2017 [17]	30 EU countries (27 EU plus Iceland, Norway, Switzerland)	Spending on Social protection as a share of GDP (EU-SILC) (2008–13)	Relative and anchored (2008) child poverty rates	Low (78.75)^a^
Gunnlaugsson 2015 [18]	Iceland	Governmental responses to the crisis (2004–14)	Social determinants and child health	Low (76.25)^a^
Herranz-Aguayo et al. 2016 [19]	Spain and Portugal	Government investment in family function (EU-SILC)	Child poverty rates and AROPE taxes	Intermediate (62.5)^a^
Nygard et al. 2019 [20]	22 EU countries	Public expenditure on family cash benefits and in-kind transfer benefits (OECD) (2006–15)	Child poverty rates (EU-SILC)	Low (87.5)^a^
Rajmil et al. 2018 [21]	16 EU countries	Countries stratified in 3 austerity groups according to the CAPB (IMF) (2005–15)	Material deprivation, child poverty, perinatal outcomes (EU-SILC), (OECD)	Low (82.5)^a^
Rajmil et al. 2015 [22]	Spain	Government responses to the crisis (2005–13)	Social determinants, child health, and HCS	Intermediate (67.5)^a^
Robinson et al. 2019 [23]	England	Effects during and after the English inequality strategy (1998–2010 / 2011–17)	IM according to the Towsend index of deprivation area in quintiles	Low (83.7)^a^
Toffolutti et al. 2018 [24]	Italy	Public health expenditure 2000–14	MMR coverage by health region	Low (82.5)^a^
Zografaki et al. 2018 [25]	Greece	Changes in perinatal outcomes centered on the long term trends (1980–2004 and 2004–14)	Perinatal outcomes at early (2008–10) and “established crisis” (2011–14)	Intermediate (67)
**Before-after approach**				
D’Agostino et al.2019 [26]	Italy, Greece, France, United Kingdom	Changes in social protection benefits (EU-SILC) (2009/14)	Monetary and non-monetary indicators of well-being	Intermediate (75)^a^
Stefansson et al. 2018 [27]	Iceland	People own assessment of their ability to make ends meet (EU-SILC) (2009/14)	Material deprivation by dimensions, vulnerability	High (46.25)^a^
**Cohort study**				
Reinhard et al. 2018 [28]	Ireland	Cohort GUI; 3 waves included a question on reduction in social welfare benefits (2009/11/13)	Family living conditions; child health, etc	Low (82.9)
**Qualitative study**				
Stalker et al. 2015 [29]	Scotland	Survey to providers for disabled children, and focus groups with carers and children (2011–13)	Changes in access and quality of services after budget cuts	STROBE: High (47.7) EPICURE (intermediate risk)

^a^Twenty was considered as the maximum score for STROBE given that a couple of items were not applicable. *AROPE* At risk of poverty and social exclusion, *CAPB* Cyclically Adjusted Primary Balance, *EU-SILC* European Union Survey on Income and Living Conditions, *GDP* Gross Domestic Product, *GUI* Growing Up in Ireland, *IMF* International Monetary Fund, *OECD* Organisation for Economic Cooperation and Development, *HCS* Healthcare services, *MMR* Measles, mumps, rubella immunisation, *STROBE* STrengthening the Reporting of OBservational studies in Epidemiology (score = 0 to 22)

A before-after approach was used to compare Italy, Greece, France and the UK [26], in terms of the ability of social benefits to reduce child poverty. One cross-sectional study collected information on a survey to families of disabled children and professionals from 32 European countries [16], while a follow-up of a cohort of children from Ireland reported on the consequences of reported reductions on welfare benefits [28], and a qualitative study from Scotland provided information on the impact of austerity measures on families with disabled children [29].

### Risk of bias

Seven out of 14 studies showed a low risk of bias according to STROBE (average score = 72.1%; median = 75.6%), 2 studies showed high risk of bias, while the qualitative study showed intermediate risk of bias on the EPICURE assessment.

### Social determinants of child health (SDCH) (Table 2)

The study analysing indicators of child poverty and material deprivation in 16 European countries according to the extent of austerity implemented by governments found that material deprivation increased in the period 2012–2015 in countries with high austerity level (interaction austerity*period 2012–15 = B: 5.62: *p* < 0.001) [21]. Children were significantly less likely to be poor in countries with higher levels of social protection spending in 2008–2013 in 30 EU countries [17]. Spending on in-kind benefits was more effective in reducing child poverty (B = − 1.6) than for cash benefits (B = − 1.2), although a gradual downward trend in the efficacy of both coefficients over time was reported [20]. Child and family poverty rates increased in Spain and Portugal following the crisis partly due to low levels of social protection; social transfers in both countries only reduced poverty and social exclusion by 7.4% [19, 22].

**Table 2:** Social determinants of child health (SDCH): child poverty, material deprivation, and social inequalities

First author (year)	Main results
Rajmil et al. 2018 [21]	Material deprivation increased during the period 2012–15 in those countries with higher austerity (interaction austerity*period 2012–15 = B: 5.62: *p* < 0.001)
Chzhen et al. 2017 [17]	Children were significantly less likely to be poor in countries with higher levels of social protection spending in 2008–2013, even after controlling for the socio-demographic structure of the population, per capita GDP and the working age unemployment rate. The effects of spending were larger and more precisely estimated for relative rather than anchored poverty, and it was not statistically significant in the last 2 years of the study (2012/13)
Nygard et al. 2019 [20]	Child poverty: the coefficients for spending on in-kind benefits (B = −1.6) were negative and consistently stronger than for cash benefits (B = − 1.2), even when controlling for other variables. There was also a gradual downward trend in the strengths of both coefficients as well as in the R Squares over time, which indicates that both forms of spending have become less efficient in reducing poverty over the studied period. This result can at least partly be attributed to higher unemployment of parents and a lower up-take rate of services (such as childcare services), as well as to cuts in the generosity of cash transfers to families
D’Agostino et al. 2019 [26]	The shares of the social benefits devoted to the Family/Children function were approximately double in the UK and France than those of Italy and Greece. The higher and lower level of expenses in family/children benefits were for Italy: 5.4% (2014) and 4.1% in 2010; Greece: 4.4% (2014) and 3.5% in 2012; France: 8.6% (2007) and 7.6% in 2014; UK: 11.3% (2010) and 10.3% in 2013.
Herranz-Aguayo et al. 2016 [19]	In 2013, Portugal exceeds the average on EU child poverty by almost 3% and Spain by almost 7% points. In Spain, one in three households is below the poverty thresholds (33.9%), followed by Portugal (31.1%). In Spain and Portugal, the ability to reduce poverty rates is much lower, remaining below the EU average (EU-15: 9.6% and EU-27: 9.1%), since both countries only achieved to reduce by 7.4% the risk of poverty and social exclusion after social transfers
Stefansson et al. 2018 [27]	Both children’s deprivation and economic vulnerability were measured at higher levels in 2014 than in 2009, though only the change in the latter was statistically significant. Rates of deprivation in individual dimensions was low and the overlap very limited, which may be indicative of low deprivation rates
Gunnlaugsson 2015 [18]	Governmental responses gave prominence to redistribution, through taxes and the social protection system. A set of measures represented protection of children were specifically improved (mental health, maternity care, immunisation, etc.). Percentage of children living in poverty almost not modified

*EU* European Union, *GDP* Gross Domestic Product

Shares of the social benefits devoted to family/children in the UK and France were approximately double those in Italy and Greece. In the study from D’Agostino et al., UK was one of the countries in which deterioration in some of the measures of well-being was avoided [26]. In Iceland, both children’s deprivation and economic vulnerability were measured at higher levels in 2014 than in 2009, though only the change in the latter was statistically significant [27]. Rates of deprivation and vulnerability were low and the overlap very limited, which may be indicative of low deprivation rates [18, 27].

### Child health outcomes (CHO)

Results on CHO are summarised in Table 3. Increasing rates of low birth weight (LBW) were associated to high level of austerity in the study of 16 countries (interaction austerity*period 2012–15, B: 0.25; *p* = 0.004) [21]. Preterm birth and LBW increased by 37 and 7% respectively in Greece the years 2011–14 [25]. In Iceland, small for gestational age increased from 2 to 3.4% [18].

**Table 3:** Child health and healthcare services

First author	Main results
**Perinatal indicators and child health**	
Rajmil 2018 et al. [21]	LBW increased during the period 2012–15 in those countries with higher austerity (interaction austerity*period 2012–15, B: 0.25; p = 0.004)
Zografaki et al. 2018 [25]	LBW increased (Standardised rate ratio, SRR = 1.07[1.06–1.09]), as well as preterm births (SRR = 1.39, [1.37–1.42] during established crisis. Some differences found according to maternal origin and age
Gunnlaugsson 2015 [18]	Governmental responses gave prominence to redistribution, through taxes and the social protection system. A set of measures protected children and were specifically improved (mental health, maternity care, immunisation). A few indicators worsened (i.e. small for gestation age changed from 2 to 3.4%.)
Robinson et al. 2018 [23]	Absolute inequalities on IMR increased in 1990–1999 (annual change between the most deprived local authorities and the rest of England = 0.03) decreased during the welfare strategy period 2000–2010 (−0.11) and increased in 2011–2017 (0.04). The analysis suggests that it is increases in public spending on healthcare and welfare that are associated with decreases in inequalities in the IMR.
Reinhard et al. 2018 [28]	48% in 2011 and 60% in 2013 reported a reduction in welfare benefits. Besides the effect of the crisis itself, it was associated with an increased risk of reporting asthma (β = 0.014, 95% CI: 0.004, 0.023) and atopy symptoms (β = 0.014, 95% CI: 0.001, 0.027).
Rajmil et al. 2015 [22]	Great impact on health of vulnerable children related to cutting budgets on housing, access to HCS, preschool investment. Increasing number of children living in poverty. No impact on child health at general population level but to the most vulnerable groups
**Mental health, and disability**	
Horridge et al. 2019 [16]	Health care professionals reported worsening quality of services than 3 years ago: increased waiting times, and less time allocated to see each child compared to 3 years ago, and worse working conditions in the last year. Nine in every ten families reported worsening quality of services for their disabled children compared to 3 years ago. Families from countries with austerity cuts reported more difficult access to welfare support and benefits.
Stalker et al. 2015 [29]	Reduction or withdrawal of services in a wide range of provision—social work, education, voluntary organisations, health and professions allied to medicine. Examples of services that were not provided or shortened. Closure of day centres. Voluntary sector survey: A Shift from Preventative Work to Crisis Intervention. Increase in unmet needs. Some families waited between one and 3 years for assessments or services on child mental health for diagnose, equipment and/or home extensions. Difficulty meeting the needs of children on the autistic spectrum was a recurring theme
**Preventive services**	
Toffolutti et al. 2018 [24]	PHE fell by 2% in the whole country between 2010 and 2014. By regions, each 1% annual reduction was associated to 0.5% (0.36–0.65) reduction on the coverage on MMR

*IMR* Infant mortality rates, *HCS* healthcare services, *LBW* low birth weight, *PHE* Public Health Expenditure

Absolute inequalities in infant mortality rates (IMR) in England increased in 1990–1999 (annual changes between the most deprived local authorities and the rest of England: 0.03), decreased during the welfare strategy period 2000–2010 (− 0.11) and increased in 2011–2017 (0.04) after the end of this strategy [23]. The study Growing Up in Ireland (GUI) found that 48% of participating families in 2011 (2y old children) and 60% in 2013 (4y olds) reported a reduction in welfare benefits, and it was associated with an increased risk of reporting asthma and atopy symptoms in the latter period [28].

### Healthcare services, and preventive services

In the study of 32 European countries, families from countries with high austerity level reported more difficulties in access to healthcare services and benefits for children with disability; professionals reported worse quality of services provided and increasing waiting time for visits [16].

Stalker et al., [29] in a study of Scotland showed a reduction or withdrawal of services in a wide range of provision—social work, education, voluntary organisations, health and professions allied to medicine. Their survey described a shift in the voluntary preventative work to crisis intervention and an increase in unmet needs. Waiting time for some families for assessments on child mental health services for diagnosis, equipment and/or home extensions were between one and 3 years. A recurring theme was the difficulty meeting the needs of children on the autistic spectrum [29].

An annual reduction of 1% in public health expenditure (PHE) was associated to 0.5% reduction in Measles-Mumps-Rubella (MMR) vaccination coverage by region in Italy [24].

## Discussion

This systematic review of studies evaluating the consequences of austerity measures on child health and well-being results support the hypothesis that those countries that have applied and maintained high levels of austerity have experienced adverse consequences for children. These consequences include deteriorating social determinants as well as child health outcomes and reduced access to, and quality of, preventive and curative healthcare services. This negative impact on child health and development may have implications for the future adult health of a generation.

This review corroborates other studies at the European general population level [1, 6]. Despite considerable cross-country differences, these studies suggest that the interaction of fiscal austerity with economic shocks and weak social protection ultimately may lead to social crisis with a negative impact on health. In countries that applied high levels of austerity, another review at the European general population level showed an increase in homelessness, food insecurity, and worsening mental health and increase of suicide rates, as well as difficulties with access to care [2]. In Greece, Spain, UK, and France charities also reported marked rises in people seeking emergency food support coinciding with the introduction of austerity measures.

The present review shows significant variability in the situation of European countries before and during the crisis. Classification of countries according to the level of austerity also shows an important variability. However, the results are consistent with respect to the impact of austerity measures on SDCH and CHO.

Poverty reduction strategies, either in-kind or cash benefits, were suggested to be less effective as a result of austerity measures employed by governments [17, 20]. Investment in family and child policies declined during the study period even in countries that traditionally invested more, and was clearly insufficient in countries such as Spain and Portugal [19, 26]. And these changes coincide again, with the period of greatest adjustment for austerity.

The impact on children’s health has been detected especially in perinatal indicators and in countries that have significantly reduced the healthcare budget, such as Greece [16]. The impact has been greater in families with vulnerable children and a very important change has been detected in the access to, and provision of, services for children with disabilities and in child mental health services. Child and adolescent mental health services in Greece operate with 10–40% fewer employees, and this situation coincides with a rise in demand for child psychiatric services but also to a qualitative change in the severity of psychopathology dealt with in everyday clinical practice [30].

Austerity measures have been reported to impact deprived groups the most [2]. In this sense, children represent a particularly vulnerable population group. Inequalities in early child development have been identified as a major contributing factor to inequalities in adult health [31]. Besides the studies included in the present review, the recent increase in infant mortality in the poorest areas of England associated with rising child poverty would also be associated to the impact of austerity policies [32], and the need to urgently address these policies for the real protection of families and children.

Although all but three of the included studies showed low risk of bias there are some limitations. First, the analysis of ecological data prevents the establishment of causal associations. Moreover, some studies comparing SDCH included ecologic exposure or outcome measures which prevents application of some items in STROBE. Nevertheless, almost all included studies show that a high level of austerity is associated with worse outcomes in SDCH. Secondly, differences in classifications used to assess the level of austerity make it difficult to establish comparisons and summary measures. Moreover, these classifications do not discriminate well between specific aspects of budget reduction, such as family benefits, early child investment, prenatal care, etc. It may not adequately reflect specific national policies that establish general economic rigor while trying to protect the most vulnerable groups, such as children. This could partly explain why Iceland [18], despite having a high level of austerity at the beginning of the economic crisis and in response to the crisis, showed trends more consistent with countries with low levels of austerity and protecting social benefits for children. This fact could be associated to a robust primary healthcare system with universal access and extensive preventive child health services, and governmental emphasis to protect low-income groups through redistribution of tax revenues and innovative labour market initiatives [33]. On the other hand, almost all included studies agree that countries such a Greece, Spain, Portugal or the UK applied higher levels of austerity than the rest. Thirdly, it was not possible to include a specific analysis of child maltreatment and trends in proportion of children in care and social protection due to the lack of reliable and comparable data at European level, and this should be the matter for future studies. Finally, there is a lack of studies of trends in gradients in social inequalities in child health, while studies at the general population level showed an increase in inequalities during the recession and austerity periods [34]. A study that analysed the investment per child in England according to the area of residence found a greater reduction in areas with greater deprivation [35]. In summary, this review highlights the need for more robust studies of the impact of austerity on child health in individual countries.

## Conclusions

Countries that applied high levels of austerity showed worse trends on SDCH, CHO, and access to, and quality of, preventive and curative healthcare services, demonstrating the importance of economic policy for equity in child health and development [36]. European governments must act urgently and reverse these austerity policy measures that are detrimental to family benefits and child protection.

## Supplementary information

**Additional file 1.**

## Data Availability

The datasets used and/or analysed during the current study are available from the corresponding author on reasonable request.

## Abbreviations

CHO: Child health outcomes
CAPB: Cyclically Adjusted Primary Balance
EPICURE: Engagement, Processing, Interpretation, Critique Usefulness, Relevance and Ethics
GDP: Gross Domestic Product
IMF: International Monetary Fund
SDCH: Social determinants of child health
STROBE: Strengthening the Reporting of Observational Studies in Epidemiology
