# Supplementary material for: Austerity policy and child health in European countries: a systematic literature review
Source: BMC Public Health. 2020 May 19;20:564. doi: 10.1186/s12889-020-08732-3 (PMC7236143; doi:10.1186/s12889-020-08732-3)
Supplement: Supplementary file 1 — Additional file 1. [file 12889_2020_8732_MOESM1_ESM.docx]

1. Medline

| Interface: Ovid MEDLINE(R) and Epub Ahead of Print, In-Process & Other Non-Indexed Citations and Daily  Date of Search: 4 November 2019  Number of hits: 437  Comment: In Ovid, two or more words are automatically searched as phrases; i.e. no quotation marks are needed | Field labels   - exp/ = exploded MeSH term - / = non exploded MeSH term - .ti,ab,kf. = title, abstract and author keywords - adjx = within x words, regardless of order - * = truncation of word for alternate endings |
| --- | --- |
| Database(s): **Ovid MEDLINE(R) and Epub Ahead of Print, In-Process & Other Non-Indexed Citations and Daily** 1946 to November 01, 2019  Search Strategy:   \| **#** \| **Searches** \| **Results** \| \| --- \| --- \| --- \| \| 1 \| exp Child/ \| 1860061 \| \| 2 \| Adolescent/ \| 1968772 \| \| 3 \| exp Infant/ \| 1113152 \| \| 4 \| Child Health/ \| 2261 \| \| 5 \| Child Welfare/ \| 21595 \| \| 6 \| Child Mortality/ \| 2373 \| \| 7 \| exp Child Health Services/ \| 23644 \| \| 8 \| Infant Health/ \| 633 \| \| 9 \| Infant Welfare/ \| 2746 \| \| 10 \| exp Infant Mortality/ \| 29487 \| \| 11 \| Adolescent Health/ \| 891 \| \| 12 \| Adolescent Health Services/ \| 5448 \| \| 13 \| (adolescen* or boy* or child* or girl* or infant* or juvenile* or minor* or neonatal* or newborn* or pediatric* or paediatric* or preschool* or toddler* or teen* or youth* or young*).ti,ab,kf. \| 2989996 \| \| 14 \| or/1-13 \| 4715216 \| \| 15 \| Economic recession/ \| 2265 \| \| 16 \| ((budget or business cycle* or economic* or financ* or macroeconomic* or socioeconomic*) adj3 (collapse* or cris* or cut* or downturn* or fluctuation* or shock*)).ti,ab,kf. \| 4940 \| \| 17 \| (2008 cris* or austerit* or recession).ti,ab,kf. \| 9506 \| \| 18 \| or/15-17 \| 14706 \| \| 19 \| 14 and 18 \| 3821 \| \| 20 \| exp Europe/ \| 1378438 \| \| 21 \| (albania* or andorra* or austria* or balkan* or baltic* or belarus* or belgi* or bosnia* or bulgaria* or croatia* or czech or danish or denmark or dutch or england or estonia* or europ* or finland* or finnish or france or french or german* or gibraltar or great britain or greece or greek or herzegovina* or hungar* or iceland* or ireland or irish or italian or italy or kosov* or latvia* or liechtenstein* or lithuania* or luxembourg* or macedonia* or moldova* or monaco* or montenegr* or netherlands or nordic countr* or norw* or poland or polish or portug* or romania* or russia* or san marino or scandinavia* or scotland* or scottish or serbia* or slovakia* or slovenia* or spain or spanish or swed* or swiss or switzerland* or ukrain* or united kingdom or wales*).ti,ab,kf. \| 1250394 \| \| 22 \| or/20-21 \| 2022787 \| \| 23 \| 19 and 22 \| 812 \| \| 24 \| limit 23 to yr="2014 -Current" \| 437 \| | |

2. Embase

| Interface: embase.com  Date of Search: 5 November 2019  Number of hits: 501  Comment: Emtree is the controlled vocabulary in Embase | Field labels   - /exp = exploded Emtree term - /de = non exploded Emtree term - ti,ab = title and abstract - NEAR/x = within x words, regardless of order - * = truncation of word for alternate endings |
| --- | --- |
| **Embase Session Results**  No.  Query  Results  501  #12  #3 AND #7 AND #10 AND [2014-2019]/py  899  #11  #3 AND #7 AND #10  2,720,407  #10  #8 OR #9  1,959,634  #9  albania*:ti,ab,kw OR andorra*:ti,ab,kw OR austria*:ti,ab,kw OR balkan*:ti,ab,kw OR baltic*:ti,ab,kw OR belarus*:ti,ab,kw OR belgi*:ti,ab,kw OR bosnia*:ti,ab,kw OR bulgaria*:ti,ab,kw OR croatia*:ti,ab,kw OR czech:ti,ab,kw OR danish:ti,ab,kw OR denmark:ti,ab,kw OR dutch:ti,ab,kw OR england:ti,ab,kw OR estonia*:ti,ab,kw OR europ*:ti,ab,kw OR finland*:ti,ab,kw OR finnish:ti,ab,kw OR france:ti,ab,kw OR french:ti,ab,kw OR german*:ti,ab,kw OR gibraltar:ti,ab,kw OR 'great britain':ti,ab,kw OR greece:ti,ab,kw OR greek:ti,ab,kw OR herzegovina*:ti,ab,kw OR hungar*:ti,ab,kw OR iceland*:ti,ab,kw OR ireland:ti,ab,kw OR irish:ti,ab,kw OR italian:ti,ab,kw OR italy:ti,ab,kw OR kosov*:ti,ab,kw OR latvia*:ti,ab,kw OR liechtenstein*:ti,ab,kw OR lithuania*:ti,ab,kw OR luxembourg*:ti,ab,kw OR macedonia*:ti,ab,kw OR moldova*:ti,ab,kw OR monaco*:ti,ab,kw OR montenegr*:ti,ab,kw OR netherlands:ti,ab,kw OR 'nordic countr*':ti,ab,kw OR norw*:ti,ab,kw OR poland:ti,ab,kw OR polish:ti,ab,kw OR portug*:ti,ab,kw OR romania*:ti,ab,kw OR russia*:ti,ab,kw OR 'san marino':ti,ab,kw OR scandinavia*:ti,ab,kw OR scotland:ti,ab,kw OR scottish*:ti,ab,kw OR serbia*:ti,ab,kw OR slovakia*:ti,ab,kw OR slovenia*:ti,ab,kw OR spain:ti,ab,kw OR spanish:ti,ab,kw OR swed*:ti,ab,kw OR swiss:ti,ab,kw OR switzerland*:ti,ab,kw OR ukrain*:ti,ab,kw OR 'united kingdom':ti,ab,kw OR wales*:ti,ab,kw  1,607,159  #8  'europe'/exp  16,404  #7  #4 OR #5 OR #6  10,324  #6  '2008 cris*':ti,ab,kw OR austerit*:ti,ab,kw OR recession:ti,ab,kw  5,696  #5  ((budget OR 'business cycle*' OR economic* OR financ* OR macroeconomic* OR socioeconomic*) NEAR/3 (collapse* OR cris* OR cut* OR downturn* OR fluctuation* OR shock*)):ti,ab,kw  2,526  #4  'economic recession'/exp  5,274,744  #3  #1 OR #2  3,837,710  #2  adolescen*:ti,ab,kw OR boy*:ti,ab,kw OR child*:ti,ab,kw OR girl*:ti,ab,kw OR infant*:ti,ab,kw OR juvenile*:ti,ab,kw OR minor*:ti,ab,kw OR neonatal*:ti,ab,kw OR newborn*:ti,ab,kw OR pediatric*:ti,ab,kw OR paediatric*:ti,ab,kw OR preschool*:ti,ab,kw OR toddler*:ti,ab,kw OR teen*:ti,ab,kw OR youth*:ti,ab,kw OR young*:ti,ab,kw  3,629,273  #1  'child'/exp OR 'adolescent'/exp OR 'child health'/exp OR 'child health care'/exp OR 'child welfare'/exp OR 'adolescent health'/exp | |

3. Web of Science Core Collection

| Interface: Clarivate Analytics  Date of Search: 4 November 2019  Number of hits: 754 | Field labels   - TS/Topic = title, abstract, author keywords and Keywords Plus - NEAR/x = within x words, regardless of order - * = truncation of word for alternate endings   Note: sometimes “quotation marks” are needed for single search terms to avoid automatic term mapping (lemmatization). |
| --- | --- |
| \| # 5 \| [**753**](http://apps.webofknowledge.com/summary.do;jsessionid=F151D37F86BD398518F429CB36F0581B?product=WOS&doc=1&qid=9&SID=C5uKWmg7FAlgEnXSsZK&search_mode=AdvancedSearch&update_back2search_link_param=yes) \| #4  *Indexes=SCI-EXPANDED, SSCI, A&HCI, CPCI-S, CPCI-SSH, ESCI Timespan=2014-2019* \|  \|  \|  \| \| --- \| --- \| --- \| --- \| --- \| --- \| \|  \| \| \| \| \| \| \| # 4 \| [**1,046**](http://apps.webofknowledge.com/summary.do;jsessionid=F151D37F86BD398518F429CB36F0581B?product=WOS&doc=1&qid=8&SID=C5uKWmg7FAlgEnXSsZK&search_mode=CombineSearches&update_back2search_link_param=yes) \| #3 OR #1  *Indexes=SCI-EXPANDED, SSCI, A&HCI, CPCI-S, CPCI-SSH, ESCI Timespan=1945-2019* \|  \|  \|  \| \|  \| \| \| \| \| \| \| # 3 \| [**945**](http://apps.webofknowledge.com/summary.do;jsessionid=F151D37F86BD398518F429CB36F0581B?product=WOS&doc=1&qid=7&SID=C5uKWmg7FAlgEnXSsZK&search_mode=AdvancedSearch&update_back2search_link_param=yes) \| TS=(adolescen* or boy* or child* or girl* or infant* or juvenile* or minor* or neonatal* or newborn* or pediatric* or paediatric* or preschool* or toddler* or teen* or youth* or young*) AND TS=(("2008 cris*" or austerit* or recession*) OR ((budget OR "business cycle*" OR economic* OR financ* OR macroeconomic* OR socioeconomic*) NEAR/3 (collapse* OR cris* OR cut* OR downturn* OR fluctuation* OR shock*))) AND TS(albania* or andorra* or austria* or balkan* or baltic* or belarus* or belgi* or bosnia* or bulgaria* or croatia* or czech or danish or denmark or dutch or england or estonia* or europ* or finland* or finnish or france or french or german* or gibraltar or "great britain" or greece or greek or herzegovina* or hungar* or iceland* or ireland or irish or italian or italy or kosov* or latvia* or liechtenstein* or lithuania* or luxembourg* or macedonia* or moldova* or monaco* or montenegr* or netherlands or "nordic countr*" or norw* or poland or polish or portug* or romania* or russia* or "san marino" or scandinavia* or scotland* or scottish or serbia* or slovakia* or slovenia* or spain or spanish or swed* or swiss or switzerland* or ukrain* or "united kingdom" or wales*)  Refined by: WEB OF SCIENCE CATEGORIES: ( CARDIAC CARDIOVASCULAR SYSTEMS OR PUBLIC ENVIRONMENTAL OCCUPATIONAL HEALTH OR ERGONOMICS OR SOCIOLOGY OR PSYCHOLOGY MULTIDISCIPLINARY OR SOCIAL SCIENCES INTERDISCIPLINARY OR OPHTHALMOLOGY OR HEALTH POLICY SERVICES OR BIOTECHNOLOGY APPLIED MICROBIOLOGY OR PSYCHOLOGY CLINICAL OR DEMOGRAPHY OR NUTRITION DIETETICS OR SOCIAL WORK OR SOCIAL ISSUES OR PSYCHIATRY OR SUBSTANCE ABUSE OR RESPIRATORY SYSTEM OR MEDICINE RESEARCH EXPERIMENTAL OR DENTISTRY ORAL SURGERY MEDICINE OR PSYCHOLOGY SOCIAL OR REHABILITATION OR ENDOCRINOLOGY METABOLISM OR CRIMINOLOGY PENOLOGY OR MEDICINE GENERAL INTERNAL OR PSYCHOLOGY OR PSYCHOLOGY APPLIED OR SOCIAL SCIENCES BIOMEDICAL OR PEDIATRICS OR CLINICAL NEUROLOGY OR IMMUNOLOGY OR NURSING OR OBSTETRICS GYNECOLOGY OR ETHNIC STUDIES OR CULTURAL STUDIES OR HEALTH CARE SCIENCES SERVICES OR BEHAVIORAL SCIENCES OR PSYCHOLOGY DEVELOPMENTAL OR MULTIDISCIPLINARY SCIENCES OR ONCOLOGY OR FAMILY STUDIES OR DERMATOLOGY OR ANTHROPOLOGY )  Indexes=SCI-EXPANDED, SSCI, A&HCI, CPCI-S, CPCI-SSH, ESCI Timespan=1945-2019 \|  \|  \|  \| \|  \| \| \| \| \| \| \| # 2 \| [**1,906**](http://apps.webofknowledge.com/summary.do;jsessionid=F151D37F86BD398518F429CB36F0581B?product=WOS&doc=1&qid=6&SID=C5uKWmg7FAlgEnXSsZK&search_mode=AdvancedSearch&update_back2search_link_param=yes) \| TS=(adolescen* or boy* or child* or girl* or infant* or juvenile* or minor* or neonatal* or newborn* or pediatric* or paediatric* or preschool* or toddler* or teen* or youth* or young*) AND TS=(("2008 cris*" or austerit* or recession*) OR((budget OR "business cycle*" OR economic* OR financ* OR macroeconomic* OR socioeconomic*) NEAR/3 (collapse* OR cris* OR cut* OR downturn* OR fluctuation* OR shock*))) AND TS=(albania* or andorra* or austria* or balkan* or baltic* or belarus* or belgi* or bosnia* or bulgaria* or croatia* or czech or danish or denmark or dutch or england or estonia* or europ* or finland* or finnish or france or french or german* or gibraltar or "great britain" or greece or greek or herzegovina* or hungar* or iceland* or ireland or irish or italian or italy or kosov* or latvia* or liechtenstein* or lithuania* or luxembourg* or macedonia* or moldova* or monaco* or montenegr* or netherlands or "nordic countr*" or norw* or poland or polish or portug* or romania* or russia* or "san marino" or scandinavia* or scotland* or scottish or serbia* or slovakia* or slovenia* or spain or spanish or swed* or swiss or switzerland* or ukrain* or "united kingdom" or wales*)  *Indexes=SCI-EXPANDED, SSCI, A&HCI, CPCI-S, CPCI-SSH, ESCI Timespan=1945-2019* \|  \|  \|  \| \|  \| \| \| \| \| \| \| # 1 \| [**586**](http://apps.webofknowledge.com/summary.do;jsessionid=F151D37F86BD398518F429CB36F0581B?product=WOS&doc=1&qid=1&SID=C5uKWmg7FAlgEnXSsZK&search_mode=AdvancedSearch&update_back2search_link_param=yes) \| **TOPIC:** (adolescen* or boy* or child* or girl* or infant* or juvenile* or minor* or neonatal* or newborn* or pediatric* or paediatric* or preschool* or toddler* or teen* or youth* or young*) *AND* **TOPIC:** (("2008 cris*" or austerit* or recession*) OR ((budget OR "business cycle*" OR economic* OR financ* OR macroeconomic* OR socioeconomic*) NEAR/3 (collapse* OR cris* OR cut* OR downturn* OR fluctuation* OR shock*))) *AND* **TOPIC:** (albania* or andorra* or austria* or balkan* or baltic* or belarus* or belgi* or bosnia* or bulgaria* or croatia* or czech or danish or denmark or dutch or england or estonia* or europ* or finland* or finnish or france or french or german* or gibraltar or "great britain" or greece or greek or herzegovina* or hungar* or iceland* or ireland or irish or italian or italy or kosov* or latvia* or liechtenstein* or lithuania* or luxembourg* or macedonia* or moldova* or monaco* or montenegr* or netherlands or "nordic countr*" or norw* or poland or polish or portug* or romania* or russia* or "san marino" or scandinavia* or scotland* or scottish or serbia* or slovakia* or slovenia* or spain or spanish or swed* or swiss or switzerland* or ukrain* or "united kingdom" or wales*) *AND* **TOPIC:** (disabled OR disabilit* OR disease* OR disorder* OR health* OR immunization* OR immunization* OR medic* OR mental* OR morbidity OR mortality OR psychiatr* OR vaccination*)  *Indexes=SCI-EXPANDED, SSCI, A&HCI, CPCI-S, CPCI-SSH, ESCI Timespan=1945-2019* \|  \|  \|  \| | |

4. Psycinfo

| Interface: Ovid  Date of Search: 5 November 2019  Number of hits: 287  Comment: In Ovid, two or more words are automatically searched as phrases; i.e. no quotation marks are needed | Field labels   - exp/ = exploded controlled term - / = non exploded controlled term - .ti,ab,id. = title, abstract and author keywords - adjx = within x words, regardless of order - * = truncation of word for alternate endings |
| --- | --- |
| Database(s): **PsycINFO**1806 to October Week 3 2019 Search Strategy:   \| **#** \| **Searches** \| **Results** \| \| --- \| --- \| --- \| \| 1 \| exp Child Care/ \| 10562 \| \| 2 \| exp child welfare/ \| 9110 \| \| 3 \| adolescent health/ \| 1428 \| \| 4 \| (adolescen* or boy* or child* or girl* or infant* or juvenile* or minor* or neonatal* or newborn* or pediatric* or paediatric* or preschool* or toddler* or teen* or youth* or young*).ti,ab,id. \| 1142442 \| \| 5 \| or/1-4 \| 1142785 \| \| 6 \| ((budget or business cycle* or economic* or financ* or macroeconomic* or socioeconomic*) adj3 (collapse* or cris* or cut* or downturn* or fluctuation* or shock*)).ti,ab,id. \| 4466 \| \| 7 \| (2008 cris* or austerit* or recession).ti,ab,id. \| 2750 \| \| 8 \| or/6-7 \| 6565 \| \| 9 \| (albania* or andorra* or austria* or balkan* or baltic* or belarus* or belgi* or bosnia* or bulgaria* or croatia* or czech or danish or denmark or dutch or england or estonia* or europ* or finland* or finnish or france or french or german* or gibraltar or great britain or greece or greek or herzegovina* or hungar* or iceland* or ireland or irish or italian or italy or kosov* or latvia* or liechtenstein* or lithuania* or luxembourg* or macedonia* or moldova* or monaco* or montenegr* or netherlands or nordic countr* or norw* or poland or polish or portug* or romania* or russia* or san marino or scandinavia* or scotland* or scottish or serbia* or slovakia* or slovenia* or spain or spanish or swed* or swiss or switzerland* or ukrain* or united kingdom or wales*).ti,ab,id. \| 392531 \| \| 10 \| 5 and 8 and 9 \| 373 \| \| 11 \| 8 and 9 \| 1853 \| \| 12 \| limit 11 to (100 childhood or 200 adolescence ) \| 199 \| \| 13 \| 10 or 12 \| 445 \| \| 14 \| limit 13 to yr="2014 -Current" \| 287 \| | |

5. Sociological abstracts

| Interface: ProQuest  Date of Search: 4 November 2019  Number of hits: 90 | Field labels   - MAINSUBJECT.EXACT.EXPLODE = exploded Subject Heading - MAINSUBJECT.EXACT = non exploded Cinahl Heading - ti = title - ab = abstract - if = keyword - NEAR/x = within x words, regardless of order - * = truncation of word for alternate endings |
| --- | --- |
| (MAINSUBJECT.EXACT("Depression (Economics)") OR ti("2008 cris*" OR austerit* OR recession* OR (budget OR "business cycle*" OR economic* OR financ* OR macroeconomic* OR socioeconomic*) NEAR/3 (collapse* OR cris* OR cut* OR downturn* OR fluctuation* OR shock*)) OR if("2008 cris*" OR austerit* OR recession* OR (budget OR "business cycle*" OR economic* OR financ* OR macroeconomic* OR socioeconomic*) NEAR/3 (collapse* OR cris* OR cut* OR downturn* OR fluctuation* OR shock*)) OR ab("2008 cris*" OR austerit* OR recession* OR (budget OR "business cycle*" OR economic* OR financ* OR macroeconomic* OR socioeconomic*) NEAR/3 (collapse* OR cris* OR cut* OR downturn* OR fluctuation* OR shock*)))  AND  (ab(albania* or andorra* or austria* or balkan* or baltic* or belarus* or belgi* or bosnia* or bulgaria* or croatia* or czech or danish or denmark or dutch or england or estonia* or europ* or finland* or finnish or france or french or german* or gibraltar or ”great britain” or greece or greek or herzegovina* or hungar* or iceland* or ireland or irish or italian or italy or kosov* or latvia* or liechtenstein* or lithuania* or luxembourg* or macedonia* or moldova* or monaco* or montenegr* or netherlands or ”nordic countr*” or norw* or poland or polish or portug* or romania* or russia* or san marino or scandinavia* or scotland* or scottish or serbia* or slovakia* or slovenia* or spain or spanish or swed* or swiss or switzerland* or ukrain* or ”united kingdom” or wales) OR ti(albania* or andorra* or austria* or balkan* or baltic* or belarus* or belgi* or bosnia* or bulgaria* or croatia* or czech or danish or denmark or dutch or england or estonia* or europ* or finland* or finnish or france or french or german* or gibraltar or ”great britain” or greece or greek or herzegovina* or hungar* or iceland* or ireland or irish or italian or italy or kosov* or latvia* or liechtenstein* or lithuania* or luxembourg* or macedonia* or moldova* or monaco* or montenegr* or netherlands or ”nordic countr*” or norw* or poland or polish or portug* or romania* or russia* or san marino or scandinavia* or scotland* or scottish or serbia* or slovakia* or slovenia* or spain or spanish or swed* or swiss or Switzerland* or ukrain* or ”united kingdom” or wales) OR if(albania* or andorra* or austria* or balkan* or baltic* or belarus* or belgi* or bosnia* or bulgaria* or croatia* or czech or danish or denmark or dutch or england or estonia* or europ* or finland* or finnish or france or french or german* or gibraltar or ”great britain” or greece or greek or herzegovina* or hungar* or iceland* or ireland or irish or italian or italy or kosov* or latvia* or liechtenstein* or lithuania* or luxembourg* or macedonia* or moldova* or monaco* or montenegr* or netherlands or ”nordic countr*” or norw* or poland or polish or portug* or romania* or russia* or san marino or scandinavia* or scotland* or scottish or serbia* or slovakia* or slovenia* or spain or spanish or swed* or swiss or switzerland* or ukrain* or ”united kingdom” or wales))  AND  (ab(adolescen* OR boy* OR child* OR girl* OR infant* OR juvenile* OR minor* OR neonatal* OR newborn* OR pediatric* OR paediatric* OR preschool* OR toddler* OR teen* OR youth* OR young*) OR ti(adolescen* OR boy* OR child* OR girl* OR infant* OR juvenile* OR minor* OR neonatal* OR newborn* OR pediatric* OR paediatric* OR preschool* OR toddler* OR teen* OR youth* OR young*) OR if(adolescen* OR boy* OR child* OR girl* OR infant* OR juvenile* OR minor* OR neonatal* OR newborn* OR pediatric* OR paediatric* OR preschool* OR toddler* OR teen* OR youth* OR young*) OR (MAINSUBJECT.EXACT("Children") OR MAINSUBJECT.EXACT("Child welfare services") OR MAINSUBJECT.EXACT("Infants") OR MAINSUBJECT.EXACT("Adolescents"))) AND ((MAINSUBJECT.EXACT.EXPLODE("Health Care Services") OR MAINSUBJECT.EXACT.EXPLODE("Health") OR MAINSUBJECT.EXACT.EXPLODE("Disorders") OR MAINSUBJECT.EXACT.EXPLODE("Child Mortality") MAINSUBJECT.EXACT.EXPLODE("Vaccination") OR MAINSUBJECT.EXACT.EXPLODE("Handicapped") OR MAINSUBJECT.EXACT("Health problems")) OR ti((disabled OR disabilit* OR disease* OR disorder* health* OR medic* OR mental* OR morbidity OR mortality OR psychiatr*)) OR if((disabled OR disabilit* OR disease* OR disorder* health* OR medic* OR mental* OR morbidity OR mortality OR psychiatr*)) OR ab((disabled OR disabilit* OR disease* OR disorder* health* OR immunization* OR immunization* OR medic* OR mental* OR morbidity OR mortality OR psychiatr* OR vaccination*)))  Applied filters: 2014-01-01 – 2019 | |
